# Multimodal CNN-DDI: using multimodal CNN for drug to drug interaction associated events

**DOI:** 10.1038/s41598-024-54409-x

**Published:** 2024-02-19

**Authors:** Muhammad Asfand-e-yar, Qadeer Hashir, Asghar Ali Shah, Hafiz Abid Mahmood Malik, Abdullah Alourani, Waqar Khalil

**Affiliations:** 1 Department of Computer Science, CoE-AI, Center of Excellence Artificial Intelligence, Bahria University, Islamabad, Pakistan; 2 Department of Computer Science, Bahria University, Islamabad , Pakistan; 3 Florida International University, Miami, USA; 4 Department of Management Information Systems and Production Management, College of Business and Economics, Qassim University, Buraydah 51452, Saudi Arabia

**Keywords:** Machine Learning Models, Neural Networks, Artificial Intelligence, Convolutional Neural Network (CNN), Drugs, Chemical biology, Drug discovery, Health care, Chemical biology, Drug discovery, Health care

## Abstract

Drug-to-drug interaction (DDIs) occurs when a patient consumes multiple drugs. Therefore, it is possible that any medication can influence other drugs’ effectiveness. The drug-to-drug interactions are detected based on the interactions of chemical substructures, targets, pathways, and enzymes; therefore, machine learning (ML) and deep learning (DL) techniques are used to find the associated DDI events. The DL model, i.e., Convolutional Neural Network (CNN), is used to analyze the DDI. DDI is based on the 65 different drug-associated events, which is present in the drug bank database. Our model uses the inputs, which are chemical structures (i.e., smiles of drugs), enzymes, pathways, and the target of the drug. Therefore, for the multi-model CNN, we use several layers, activation functions, and features of drugs to achieve better accuracy as compared to traditional prediction algorithms. We perform different experiments on various hyperparameters. We have also carried out experiments on various iterations of drug features in different sets. Our Multi-Modal Convolutional Neural Network - Drug to Drug Interaction (MCNN-DDI) model achieved an accuracy of 90.00% and an AUPR of 94.78%. The results showed that a combination of the drug’s features (i.e., chemical substructure, target, and enzyme) performs better in DDIs-associated events prediction than other features.

## Introduction

A DDI occurs when two or more drugs are taken together and result in adverse effects on the organism. It is feasible to manage more drugs, but some diseases are complex and can be treated with only one. This is because many diseases are justified by multiple medications. Therefore, DDIs pose risks and can be deadly if not treated properly. However, it also has curative properties. These risks have been identified in many studies, including research aimed at identifying whether the intake of two or more drugs will be safe. In pharmaceutical research/settings, DDIs are identified through thorough experimental and clinical testing. Despite high-throughput methods, the sheer number of DDIs makes experimental testing challenging and expensive ?. Computational methods can be used to address this problem by predicting potential DDIs, which can be an effective and fast alternative based on already-known knowledge of DDIs. In the last few decades, the speedy growth in drug development has provided medical practitioners with additional options for treating the diseases of patients. Therefore, using multiple drugs together can lead the patient to a severe condition, or it can be a cause of death. A doctor or physician prescribing multiple drugs to a patient may cause a drug-to-drug interaction, or we can say the drug-to-drug interaction is a medication error^1^. A drug interaction can occur when one drug changes the action of other drugs, which may result in harmful or boost effects of the second agent (i.e., drug)^2^.

There are three types of drug-to-drug interactions.

- Does not react.
- Antagonistic (i.e., it is a type of interaction between multiple drugs that produces an adverse effect). This means that this type of reaction may adversely affect the patient.
- Synergistic (i.e., this type of interaction occurs between multiple drugs which may boost the effect on the body)^3–5^.

Therefore, to improve the drug discovery process and patient recovery process for deadly diseases (for example, Cancer, Aids, Asthma, etc.), it is significant that we should know more about DDI. Discovering the DDI’s through the wet lab experiments is time-consuming and intensive work required^6^. Drug interaction prediction usually seeks to detect potential interactions between drugs that may lead to adverse effects or reduced efficacy^7^. While Drug combination prediction is intended to improve treatment effectiveness, drug interaction prediction identify possible side effects or difficulties that may arise from the use of particular drugs in combination^8^.To overcome the above problems ML and DL techniques are applied to predict DDI. Machine learning and deep learning have made important developments in the last few years in predicting DDIs. These methods are proposed for identifying latent DDIs between drug combinations. Yet, the majority of these methods limit themselves to using drugs as input and performing the DDI prediction task. We use multiple features of drug targets, enzymes, pathways, and chemical substructures for our experiments; and we calculate similarities of drugs using Jaccard similarity measures. Our proposed method is MMCNN-DDI for the DDI’s. It is based on a Multi-Modal Convolutional Neural Network for the event prediction between DDIs. Therefore, we use four CNN sub-models for each feature of drugs and then combine these sub-models to predict drug-drug interaction events. We used DDIs using Multi-Modal Deep Learning (b) to create a dataset for the prediction; the data set has 572 drugs and various features like chemical substructures (SMILES), enzymes, pathways, and targets, 74,528 interactions, and 65 types of drug-drug interaction events. We perform various experiments on different hyperparameters. Initially, we used a different number of layers and activation functions using the dense layer and input layer according to the Jaccard similarity. We used a 1D CNN input layer with a filter size of 1 and 5 kernels, three dense layers of 1024, 512, and 256 neurons, and an output layer of 65 neurons. Our MCNN-DDI model reached an accuracy of 90.00% and an AUPR of 94.78%. The experimental outcomes show that our method has achieved high accuracy and performs well against existing methods.

## Related work

In the past decade, scientists have used ML and DL for drug development purposes to make drug development quick and help the drug industries develop drugs quickly because using wet-lab experiments is time-consuming and expensive, as discussed in some studies. The research^9^ proposed a method named Semi-Nonnegative Matrix Factorization (DDINMF) for degressive and enhancive calculation of DDI’s, and the proposed model is created on semi-non-negative matrix factorization. The study^10^ integrated various structures of drugs like chemical substructure, enzymes, pathways, and targets and proposed a method named Sparse Feature Learning ensemble method with Linear Neighborhood Regularization (SFLLN) for predicting DDI’s. Authors^11^ integrate three different deep learning techniques, which are Recurrent Neural Networks (RNNs), CNNs, and Mixture Density Neural Networks (MDNs), for the efficient prediction of DDI. The research^12^ proposed a novel method named Deep Predictor (DPDDI) for the DDI prediction in which Graph Convolutional Network (GCN) is used for the extraction of network structure features of medicine from the network of DDI’s, and a model of Deep Neural Network (DNN) is working as a predictor. Authors^13^ developed a new model that uses deep feed-forward networks and autoencoders and trained on three different similarity profiles, which are Target, Gene, and Similarity Profiles, Gene Ontology term similarity profiles, and SSP for predicting DDI’s effect.

Study^14^ proposed a method named the Neural Network-Based Method for DDIs (NDD). The model first calculates the diverse similarity of drugs, for example, pathways, side effects, target, transporter, and substructure, and then uses neural networks to predict DDIs. In 2019 authors^15^ used multi-modal deep autoencoders, which learn from multiple drug feature networks simultaneously a unified representation of drugs. Several operations are adopted on drug embedding, which is retained for the drug-drug pairs representation and then used in the random forest (RF) for the DDI’s prediction. The study^16^ use multiple drug data sources like Drug Bank^17^, KEGG drugs^18^, and PharmGKB^19^ and use 12000 features of drugs and integrate these features using Knowledge Graphs (KGs). Different embedding approaches are used to train the prediction model, leading to a best-performing combination: The ComplEX embedding method. Xinyu et al.^20^ generated features of 5000 drugs from a drug bank database and built a DNN model to predict 80 types of DDI’s using 5000 drug features; these features of drugs were produced using SMILES. Deng et al.^21^ used a drug bank database and collected DDI from that and then used event trimming and dependency analysis to extract 65 different categories of DDI and then proposed a novel framework named Drug-Drug Interaction Multimodal Deep Learning (DDIMDL), which used a sub-model for every feature of drugs and then concatenated the sub-models for the prediction of the DDI. This research^22^ used a One-Class Support Vector Machine (OCSVM) for reliable negative seed generations, then used all the positive and negative labels for training and an iterative Support Vector Machine (SVM) to identify all negative from and un-label samples to predict DDIs. The study^23^ explored graph outcomes knowledge for the DDI prediction to overcome the following two problems: the first is to achieve a good performance, and the second is to keep certain interpretability.

In this research authors^24^ created a DDI prediction framework based on Knowledge Graph (KG) embeddings and introduced a Gumbel-SoftMax and Wasserstein Distances-Based Adversarial Autoencoders (AAE’s) where the autoencoder is used to make high-quality negative samples. In 2020 study^25^ extracted various features of drugs which include targets, categories, enzymes, and pathways, focused on 65 types of DDI events, and proposed a CNN-based model named CNN-DDI for the drug-drug interaction prediction. Study^26^ introduces a multi-scale feature fusion method to fuse multi-modal features well using scalar and cross-level components. In research authors^27^ proposed a technique called Knowledge Graph Neural Network (KGNN) for the DDI’s prediction. This method learns for each entity from neighborhoods, and then the neighborhood’s information is integrated with bias from the current entity representations. Study^28^ used drug embedding and Graph autoencoders with multiple knowledge sources to effectively predict DDI’s. To learn the embeddings of drugs, they used a Drug Target Interaction network and a variational autoencoder to gain rich chemical structure representation.

In this research authors^29^ used seven types of drug pair similarities to create feature vectors and then proposed a model based on logistic regression for DDI predictions. the research^30^ and^31^ used interaction profile fingerprint and structural similarity for the DDI’s prediction. Most previous studies focuOne-Classher two drugs interacting with each other or not, and now most researchers focus on the deep learning prediction model using deep neural networks; compared with simple CNN and DNN, the multi-modal CNN performed well, which can also effectively overcome the overfitting problem as compared to simple DNN. This study developed a novel method based on a Multi-Modal CNN named (MMCNN-DDI). First, four features of drug targets, enzymes, pathways, and chemical substructures. Then we use different similarity measures like the Jaccard and Cosine similarity matrix to calculate drug pairs’ similarities. Then construct four sub-models based on a CNN for each feature of drugs and feed these similarities matrix to sub-models last; these sub-models are concatenated for predicting DDI’s. The study^32^ presents DANN-DDI, a deep attention neural network framework to predict unknown DDIs by integrating multiple drug features. The model uses a graph representation learning approach to obtain medicine embeddings, followed by an attention neural network (ANN) to acquire representations of drug-drug pairs. The technique outperforms several state-of-the-art prediction methods and can recognize innovative connections and DDI-associated events. This study^33^ presents a new method called DDI-IS-SL for predicting drug-drug interactions created on combined similarity and semi-supervised learning (SSL). DDI-IS-SL integrates drug biological, chemical, and phenotype data to compute drug similarity and uses the Regularized Least Squares classifier to predict interaction possibility scores of drug pairs. The paper^34^ discusses DDIs which occur when drugs affect each other, leading to unexpected or severe side effects. DDIs are important to consider for drug-related studies, for example, drug repurposing, and drug-target interaction. The paper introduces DDIPred, a new technique for DDI prediction that utilizes drug chemical building embedding and graph convolutional networks (GCNN). In this study^35^, a new single-stage finder model has been developed. The model comprises a base network, which is then followed by several multiscale feature map blocks. This design allows for the output of the base network to be transformed into larger feature maps, which in turn generates more anchor boxes to detect smaller objects. As a result, the size of the feature maps is decreased, allowing for more precise detection of labeled objects. This paper^36^ discusses the use of DL and ML algorithms to predict (DDIs), which is a low-cost and real method. The paper also highlights the need for further research in this area to reduce the number of interactions and their adverse effects. The recent use of deep learning techniques for recognizing relations among different medicines to evade adverse effects is also discussed, and the importance of increased accuracy and performance in predicting DDIs is emphasized. The paper concludes by suggesting that future research should consider drug-food interactions in addition to DDIs.

This research^37^ Predicting multiple interactions that a drug may encounter is crucial for drug development and safety. Artificial Intelligence (AI) has offered innovative methods to predict these interactions efficiently compared to traditional labor-intensive approaches. This research systematically examines AI applications in predicting drug-drug, drug-food (excipients), and drug-microbiome interactions. It outlines common model methods, evaluation indicators, algorithms, and databases used for these interactions. Particularly, ML models focusing on metabolic en- zyme P450, drug similarity, and drug targets are discoursed. The research^38^ study outlines progress in AI for each type, summarizing data sets and methods. It introduces common databases, presents research advancements, and traces the timeline of DDI prediction events. The paper also discusses the challenges and potential of AI in enhancing clinical decision-making and patient outcomes in DDI prediction.

In this research^39^ authors employ machine learning to predict drug risk levels based on Adverse Drug Reactions (ADRs). Using a dataset of 985,960 ADR reports from the Chinese spontaneous reporting database, we address class imbalance with the Synthetic Minority Oversampling Technique (SMOTE). The approach involves a multi-classification framework, utilizing ADR signal values and four different classifiers. The optimal combination, PRR-SMOTE-RF, achieved an accuracy rate of 95%. This study has potential applications in assisting experts in assessing the transition of prescription drugs to over-the-counter status. In this study^40^ the authors employ artificial neural networks and factor propagation over graph nodes, presenting two innovative methods: adjacency matrix factorization (AMF) and adjacency matrix factorization with propagation (AMFP). These findings emphasize the potential of AMF, AMFP, and the ensemble-based classifier in providing vital information for drug development and prescription, even with partial or noisy data. The study also underscores the importance of the drug interaction network as a valuable data source for identifying potential DDIs.

This study^41^ addresses the importance of predicting interactions between G protein-coupled receptors (GPCRs) and drugs, a crucial aspect of drug development. The results demonstrate improved predictive performance compared to existing models, offering potential benefits for drug development efforts. The study^42^ focuses on Herbs and their partnership with medicines which become popular worldwide. This study collects all the facts about Panax notoginseng and medicines, helping doctors and patients make better choices for their health.

The research^43^ examined, a novel ensemble neural network model, proposed to improve the accuracy of predicting drug-drug interactions. In this study, the authors introduce a super-smart computer model that can predict interactions between 86 different drugs with almost 94% accuracy. A comparative analysis table of the discussed work is presented in Table 1.

**Table 1:** Comparison with the related work.

Refs.	Problem statement	Models	Techniques	Results
Shukla et al.^11^	To overcome over-fitting and predict DDI using DL	(CNN), (RNN), (MDN), (LSTMRNN)	Integrated CNN, RNN, and MDN for Cancer cells	ACC of 98.4%
Feng et al.^12^	DDI prediction without using various drug properties	(GCN), (DNN), (5-fold CV)	Single feature (i.e., SMILES) of drugs	ACC of 94.0%
Lee et al.^13^	Decreasing profile size for accuracy	(Autoencoders), (Feed Forward DNN)	Autoencoders vs. PCA	ACC of 94.0%
Rohan et al.^14^	Combining similarity and NN for accuracy	(Neural networks), (GIP), (SNF), (5-fold CV)	Neural networks with similarity integration	AUC of 99.2%
Liu et al.^15^	Capturing nonlinear network structures	(DNN), (Deep Autoencoders), (3, 5-fold CV)	Random forest training	AUC of 87.03%.
Karim et al.^16^	Using data sources to predict DDIs	(Convolutional LSTM), (Knowledge Graphs), (5-fold CV)	Cov-LSTM for DDI prediction	AUPR of 93.0%.
Hou et al.^20^	Predicting DDIs using drug bank data	(DNN), (SVM)	SMILES only for DDI prediction	AUC 94.2%.
Deng et al.^21^	Predicting DDIS-associated events	(DNN), (Stanford NLP), (Similarity Matrix), (5-fold CV)	DNN for DDI’s	AUC of 88.5%.
Zheng et al.^22^	Predicting DDIs using classifiers	(SVM), (OCSVM), (KNN), (3, 5-fold CV)	SMILES with side-effect and Indication	F1-score 86.0%.
Chen^23^	Testing pairwise information similarity	(Siamese GCN), (Single Layer NN)	SMILES of drugs	F1 67.31% on large data-set
Dai^24^	Generating severe adverse effects of DDIs	(Autoencoder), (KG embedding), (Wasserstein distance), (GumbelSoftmax relaxation)	Network feature set with KG and Autoencoders	PR-AUC 76.0%.

The goal of this study^44^ is to give a comprehensive review of the current status and trends in drug-target interaction prediction. It lists several databases and web servers with data on drug space, target space, the drug-target interaction network, and side effect networks. The paper^45^ deals with the function of small molecules and microRNAs (miRNAs) in cellular biology. The authors cover four experimental methods that have been employed in the last few years to look for small molecule inhibitors of miRNAs as well as three classes of models that can be used to predict whether a compound binds with a certain miRNA. The study^46^ calls for more effective drugs to combat complex human diseases. The paper explains the background of the drug and introduces the concept of drug-pathway associations. The authors^47^ present a Multi-Channel Feature Fusion model for multi-typed DDI prediction (MCFF-MTDDI). They extracted drug chemical structure features, drug pairs’ extra label features, and KG features of drugs. A multi-channel feature fusion module was then used to fuse these various features. The study^48^ compared ML and PBPK models in predicting drug-drug interactions (DDIs). Data-driven in nature, and able to handle huge datasets with complex relations, ML models are thus well suited for predicting DDIs from disparate databases. The research proposed an integrated approach that combines ML with PBPK models, thus increasing the accuracy and efficiency of DDI predictions while also making the process more interpretable.

**Figure 1:**
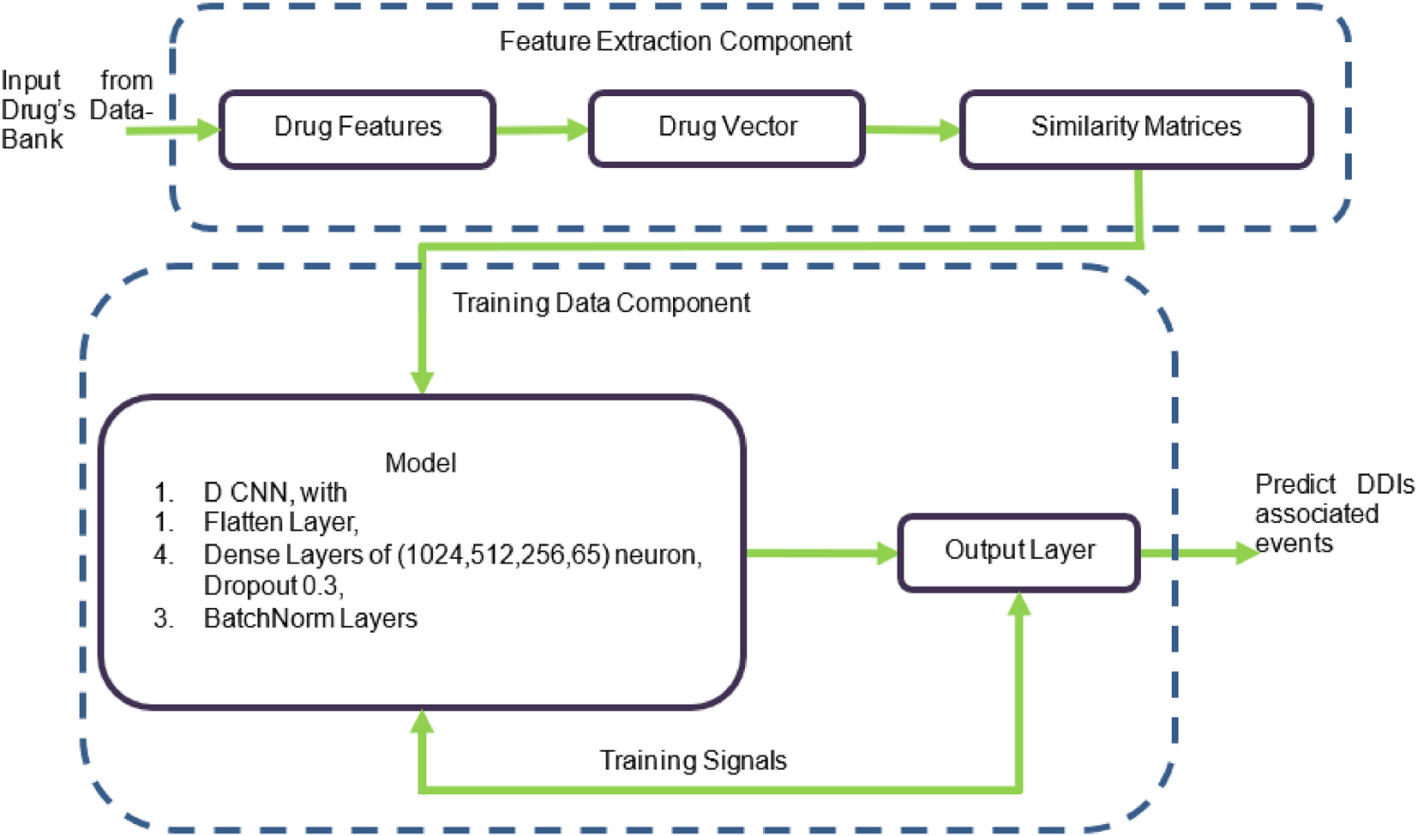
Framework architecture of DDI’s events.

## Materials and methods

The dataset used in this study was provided by the DDIMDL^21^ this dataset has 572 drugs and 74528 interactions of drug pairs according to DDIMDL. Remove^21^ the repeated interaction of drug pairs then in the dataset 37264 are left. They collected DDI’s from drug bank which was in the descriptive format and applied NLP techniques for a better understanding of DDI’s. Then four different features of drug targets, enzymes, pathways, and chemical substructures from the Drug Bank database. Drug Bank^17^database contains 12,151 drugs and its broad information which includes the drug name, chemical substructure (i.e., SMILES) or we can say the chemical formula of drugs, targets, enzymes, pathways, description, protein, etc., also contains 3844 drugs approved from Food and Drug Administration (FDA) and 5867 experimental drugs.

**Figure 2:**
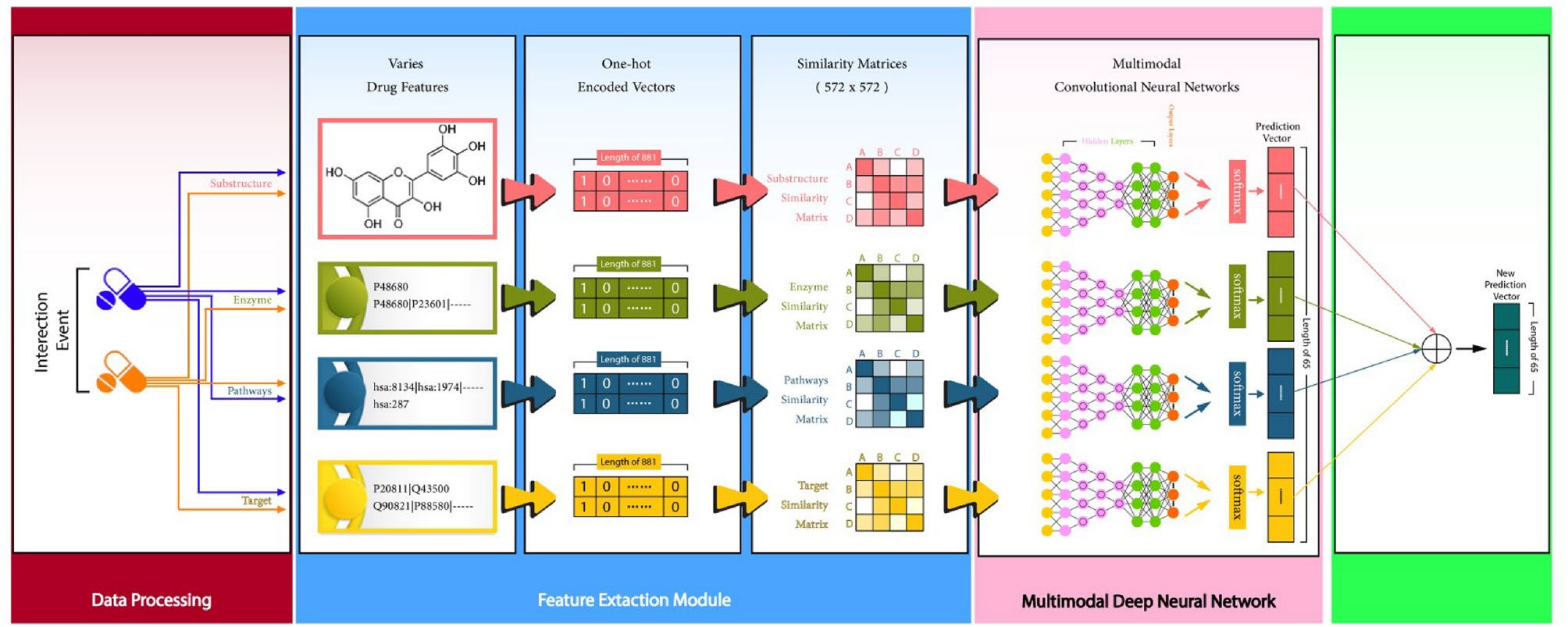
The Proposed model is named MCCN-DDI.

### Methods overview

Our proposed framework, as shown in Fig. 1, has two components. Initially, features are extracted then pass the required data in the models, to train the model. Only required preprocessing techniques are applied in the feature extraction component. Then we used feature engineering techniques on the drug’s data. In the second component, we applied the CNN to train our model and evaluated that model with multiple performance measures, for example, accuracy, precision, recall, and F1 score. In this study, we proposed a method-based multi-modal CNN for the DDIs associated events predictions, as shown in Fig.2. First, we input four features of drug targets, enzymes, and pathways Schemes follow the same formatting. If there are multiple panels, they are listed below:

- First, we input four features of drug targets, enzymes, pathways, and chemical substructure.
- Second we use encoding and get binary vectors of features where 1 represents the presence of a compound and 0 represents the absence of a compound in the drug and then use similarity measure to calculate the similarity matrix of each drug pairs.
- In the third step we create sub-models based on CNN for the prediction of DDI’s events.

### Similarity of drug pairs

For drug pairs similarity calculation, we use the Jaccard similarity in our proposed architecture. The Jaccard similarity compared a compound of two drugs to check whether the compound is shared or not. Mathematical formulas of the Jaccard similarity measure are given in Eq. (1). Jaccard Similarity Formula

1 $$\begin{aligned} J_{\text {sim}}(J, K)= & {} \frac{|J \cap K|}{|J \cup K|} \nonumber \\= & {} \frac{\text {Total number of compounds in drug pairs}}{\text {Number of compounds in either set}} \end{aligned}$$

Where:

- *J* is the bit vector for the first set (drug pair)
- *K* is the bit vector for the second set (drug pair)
- $|J \cap K|$ represents the intersection of *J* and *K*
- $|J \cup K|$ represents the union of *J* and *K*

**Table 2:** MCNN-DDI results on different layers and activation function with Jaccard Similarity.

Layers	I-Fun	D-Fun	Similarity Matrices	ACC	F1	AUC	AUPR
2	tanh	tanh	Jaccard	0.7631	0.6222	0.9664	0.8267
3	tanh	tanh	Jaccard	0.8826	0.7767	0.9979	0.9338
3	sigmoid	sigmoid	Jaccard	0.4166	0.4371	0.9664	0.3904
3	tanh	elu	Jaccard	0.8963	0.8086	0.9981	0.9449
3	tanh	relu	Jaccard	0.7952	0.5440	0.9961	0.8636
4	tanh	relu	Jaccard	0.8830	0.7779	0.9978	0.9344
4	tanh	elu	Jaccard	0.9000	0.8286	0.9981	0.9478

**Table 3:** MCNN-DDI results on different sets of features.

Set of Features	Similarity Matrices	ACC	F1	AUC	AUPR
S	Jaccard	0.8861	0.8099	0.9983	0.9505
T	Jaccard	0.8441	0.7517	0.9977	0.9254
E	Jaccard	0.6802	0.4584	0.9920	0.7691
P	Jaccard	0.8317	0.7357	0.9975	0.9169
S + T	Jaccard	0.8986	0.8264	0.9985	0.9549
S + E	Jaccard	0.8933	0.7943	0.9976	0.9309
S + P	Jaccard	0.8993	0.8294	0.9986	0.9552
T + P	Jaccard	0.8509	0.7667	0.9980	0.9313
T + E	Jaccard	0.8658	0.7498	0.9972	0.9158
P + E	Jaccard	0.8635	0.7563	0.9973	0.9160
S + P + E	Jaccard	0.8998	0.8102	0.9981	0.9464
S + T + E	Jaccard	0.9000	0.8286	0.9981	0.9478
S + T + P	Jaccard	0.8849	0.8077	0.9986	0.9530
T + P + E	Jaccard	0.8672	0.7833	0.9976	0.9292
S + T + E + P	Jaccard	0.8953	0.8164	0.992	0.9503

### Extracting drug features

We have four different drug feature which includes targets, enzymes, pathways, and chemical substructures, as shown in the layer of “Drug Features” in Fig. 1. We used the encoding layer to create a bit vector of each drug where 1 represents the presence of the compound in the drug and 0 represents the absence of the compound. For example, pathways can be represented by a 957-dimensional bit vector which is defined by the PubChem chemical molecule database^49^. PubChem defines a 202-dimensional bit vector for enzymes, an 1162-dimensional bit vector for targets, and an 808-dimensional bit vector for chemical substructure. The selected four drugs’ features for the experiment have high dimensions and most of the values are 0 in every bit vector of the drug. The value is 0 if a chemical compound is missing in a drug. Due to a maximum number of 0’s the dimension of a drug vector increases. Therefore, to reduce the drug vector sparsity we use Jaccard similarity measures. This similarity measure calculates a drug pair similarity matrix from a bit vector. Hence, we get 572 $\times$ 572 matric for each of the four drug features. This metric is used as a representation of drugs.

Let M = (Ajk) where M is the metric A is a drug and j and k are in the range of 0 and 1. Therefore, when the j and k are higher then the similarity between pairs of drugs is higher. Afterward, we feed these 572 $\times$ 572 as input of every drug feature to the sub-model based on CNN.

### Multimodal CNN for prediction

As we are using different drug features in our study, we created sub-models based on convolutional neural networks for every feature of drugs.

CNN is a type of deep neural network^50^. CNN was developed in the mid-1980s^51^. The CNN consists of three types of layers first one is input layers, second is hidden layers, and in the last output layers. The hidden layer in CNN includes layers that perform convolutions. Commonly the hidden layer of CNN includes a layer that performs convolution kernel dot product with the input matrix layer’s commonly used activation function is ReLU^52^ and usually the product is Frobenius inner product. Features maps are generated by the convolutional operations which act as an input to the next layer and then followed by other layers like normalization layers, fully connected layers, and pooling layers.

Our model architecture was inspired by DDIMDL which uses a simple multi-modal deep neural network for DDI prediction and achieved an accuracy of 88.5%.

In the CNN model, we use the input layer with the filter size of 1 and the kernel size of 5 with the “tanh“ activation function. We use the “tanh“ as an activation function because the similarity matrix using the Jaccard similarity measures produces some negative values, therefore we use “tanh” to use both positive and negative values. Then we use the flattening layer to convert the data into a dimensional array for inputting to the next layer. After flattening layer uses a dense layer of 1024,512,256 neurons and uses an “elu” as an activation function^53^. With every dense layer, we use the “Bach Normalization” layer^54^ for the normalization of the previous output layer. To avoid over-fitting of the model we also use a drop-out layer^55^ of value 0.3 with every dense layer. Then we use a dense layer of 65 neurons as an output layer because we are classifying 65 different types of drug-drug interaction with the “SoftMax” activation function. We use Adam^56^ as an optimizer in our model, and for the loss function, we use categorical cross entropy^57^.

## Results

In this section, we delve into the methods we employed and the exciting results we obtained in our study on drug-to-drug interactions (DDIs). Our approach harnessed the power of machine learning, specifically utilizing a Convolutional Neural Network (CNN) to analyze 65 unique drug-associated events extracted from the DrugBank database. Our model considers a wide range of input data, including chemical structures represented as SMILES, information about enzymes, pathways, and drug targets. Through extensive experimentation, where we fine-tuned various model parameters and explored different combinations of drug features, our Multi-Modal Convolutional Neural Network-Drug to Drug Interaction (MCNN-DDI) yielded impressive results.

Furthermore, we have carried out two different statistical tests to evaluate the performance of different methods. A paired t-test is used to compare our proposed method with other methods. This test determines if the mean difference in performance metrics is significant. For example, accuracy or AUC-ROC statistics between our method and baseline methods, the t-test shows that our proposed methods perform better than other methods, as shown in Table 4. The second ANOVA statistical test determines the effect of performance on dependent variables and improvement in evaluation metrics. A significance level $\alpha = 0.05$ was used to perform both tests. The statistical significance is indicated by the p-value if it is less than $\alpha = 0.05$.

### Performance of model on different hyperparameters

The hyperparameters can affect the performance of the model. Hence, we perform different experiments on different hyperparameters. First, we used different numbers of layers and different activation functions in the dense layer and input layer with the Jaccard similarity matrices the number of layers and the activation function results with Jaccard similarity are given in Table 2.

### Evaluating MNN-DDI performance on different set of features

For the results, we generate a classification report in our study, and the evaluation measures include accuracy score, F1-score, precision, and recall on both content and context results. All the above measurements are used for the model results evaluations. We have also done some experiments with different sets of drug features to evaluate our model performance on individual drug features and a combination of different drug features. The results on a single and a set of features are given in Table 3. The chemical substructure achieves an accuracy of 0.8861 which is more informative as compared to other features while the pathways achieve an accuracy of 0.8317 and the model train on target achieves an accuracy of 0.8441. The enzyme features of drugs individually achieve an accuracy of 0.6808 which show that this feature of drugs is not informative as compared to other. When used individually in combination with other features it produces very good results. Whenever using two sets of features the smiles and pathways achieve an accuracy of 0.8993 which is greater than all of the other drug’s features used in a set of two. Using three sets of features among all the features the following features chemical substructures, Targets, and Enzymes achieved an accuracy of 0.9000. The model is trained on all four combined features and achieved an accuracy of 0.8953. All the experiments show that using all four features of drugs combined can’t perform as well as the above three features perform.

Evaluating the performance of our model more in-depth, we investigated how often DDIs among the top 100 most highly scored predictions were correctly predicted. Therefore, we used a ranking strategy, to analyze how well our model prioritizes true positives. Hence, methodologically, we computed prediction scores for all the DDIs and ranked them based on these predictions. So, we analyze the precision predictions between different drug interaction’s ranking particularly in the best 100. By this, we learned more about the model’s ability to identify the most pertinent DDIs. Our model has demonstrated the ability to prioritize DDIs accurately as per our expectations from this top-best 100 analysis. Our model shows good performance in predicting interactions between different types of drugs such as cardiovascular, infectious, and cancerous diseases. In addition, our results were compared with the other approaches and they confirmed the effectiveness and reliability of our model to rank DDIs.

We also examined whether the model provided equal accuracy of prediction DDIs across cardiovascular, infectious, and cancer diseases. After a detailed evaluation, we noted a consistency in the performance of our model within the cardiovascular, infectious, and cancer diseases. The consistency shows the strength of our model. Our model consistently placed true positive interactions among the first-ranked predictions, indicating its capability to accurately detect clinically relevant DDIs.

## Discussion

To show the robustness of our model MMCNNDDI we compare our model (as shown in Fig. 1) with the following models which are DDIMDL, DeepDDI, RF, K-nearest Neighbor (KNN), and Logistic Regression (LR). All the given methods use using same drug features like targets, enzymes, pathways, and chemical substructure except DeepDDI (as shown in 2). For the random forest, we set the decision tree values to 100 and for KNN we set the neighbor value to 4. The experiment results of all the methods are shown in the Table 4.

**Table 4:** MCNN-DDI results comparison with other models.

Method	ACC	F1	AUC	AUPR
MCNNDDI	0.9000	0.8286	0.9981	0.9478
DDIMDL	0.8852	0.7585	0.9976	0.9208
DeepDDI	0.8371	0.6848	0.9961	0.8899
RF	0.7775	0.5936	0.9956	0.8349
KNN	0.7214	0.4831	0.9813	0.7716
LR	0.7920	0.5948	0.9960	0.8400

In Table 4, it can be observed that our model outperforms all the other models in the four assessments, while the LR model is poorly related to all the other models. Our model achieves high accuracy and AUPR values of 0.9000 and 0.9478, respectively, demonstrating its superiority (as shown in Table 3). When compared to DDIMDL, our model shows an improvement in accuracy score from 0.8852 to 0.9000 and an increase in AUPR value from 0.9208 to 0.9478, along with improvements in other evaluation metrics.

## Conclusion

Recently deep learning techniques have been used for the prediction of drug-drug interactions but mostly these studies concentrate on one feature of drugs or whether one drug interacts with another or not. In this research study, we use deep learning multi-modal CNN techniques on the drug bank database which was created by DDIMDL for the prediction of drug-drug interaction events. The data set has 572 drugs and their diverse features like chemical substructures (SMILES), enzymes, pathways, and targets, 74528 interactions, and 65 types of drug-drug interaction events. Our MCNN-DDI model achieved an accuracy of 90.00% and an AUPR of 94.78% as shown in Table 3. Our MCNN-DDI model has a 1D CNN input layer with a filter size of 1 and 5 kernels size, three dense layers of 1024, 512, and 256 neurons and an output layer of 65 neurons, 1 Flatten layer, and Bach Norm and Dropout layer with 0.3 value and use a combination of four drug features for the input of the model. We used four CNN sub-models for every feature of drugs and then in the last, we combined these sub-models for the prediction of drug-drug interaction events.

## Data Availability

All data generated or analyzed during this study are cited in Reference^19^ and are also publicly available:https://go.drugbank.com/.
